# Synthesis of Caffeoyl-Prolyl-Histidyl-Xaa Derivatives and Evaluation of Their Activities and Stability upon Long-Term Storage

**DOI:** 10.3390/ijms22126301

**Published:** 2021-06-11

**Authors:** Hyeri Jeong, Young-Eun Jeon, Jin-Kyoung Yang, Jaehi Kim, Woo-Jae Chung, Yoon-Sik Lee, Dong-Sik Shin

**Affiliations:** 1 Department of Chemical and Biological Engineering, Sookmyung Women’s University, Seoul 04310, Korea; wpg@sookmyung.ac.kr (H.J.); jun24600@sookmyung.ac.kr (Y.-E.J.); 2 School of Chemical and Biological Engineering, Seoul National University, Seoul 08826, Korea; yjk0627@snu.ac.kr (J.-K.Y.); susia45@snu.ac.kr (J.K.); 3 Department of Integrative Biotechnology, Sungkyunkwan University, Suwon 16419, Korea; wjchung@skku.edu; 4 BeadTech Inc., 10-dong 4th, 49 Wonsi-ro, Danwon-gu, Ansan-si 15610, Korea; 5 Industry Collaboration Center, Sookmyung Women’s University, Seoul 04310, Korea

**Keywords:** solid-phase synthesis, caffeic acid, caffeoyl peptide derivatives, antioxidant, stability

## Abstract

Antioxidants play a critical role in the treatment of degenerative diseases and delaying the aging of dermal tissue. Caffeic acid (CA) is a representative example of the antioxidants found in plants. However, CA is unsuitable for long-term storage because of its poor stability under ambient conditions. Caffeoyl-Pro-His-NH_2_ (CA-Pro-His-NH_2_, CA-PH) exhibits the highest antioxidant activity, free radical scavenging and lipid peroxidation inhibition activity among the histidine-containing CA-conjugated dipeptides reported to date. The addition of short peptides to CA, such as Pro-His, is assumed to synergistically enhance its antioxidative activity. In this study, several caffeoyl-prolyl-histidyl-Xaa-NH_2_ derivatives were synthesized and their antioxidative activities evaluated. CA-Pro-His-Asn-NH_2_ showed enhanced antioxidative activity and higher structural stability than CA-PH, even after long-term storage. CA-Pro-His-Asn-NH_2_ was stable for 3 months, its stability being evaluated by observing the changes in its NMR spectra. Moreover, the solid-phase synthetic strategy used to prepare these CA-Pro-His-Xaa-NH_2_ derivatives was optimized for large-scale production. We envision that CA-Pro-His-Xaa-NH_2_ derivatives can be used as potent dermal therapeutic agents and useful cosmetic ingredients.

## 1. Introduction

Reactive oxygen species (ROS) include highly reactive free radicals, such as peroxide (ROO^∙^) and superoxide (O_2_^∙^). ROS induce oxidative stress in the human body via radical chain reactions, thereby causing damage to DNA or RNA, cancer, chronic disease and aging [1,2]. In addition, oxidative stress can cause degenerative diseases such as Alzheimer’s disease [3,4,5].

Natural antioxidants found in various foods, including vegetables, meat, fruits and grains, inhibit the activity of ROS. They are considered suitable agents for scavenging radicals but are unsuitable for practical use as antioxidative nutrients or therapeutic agents because of their low concentration and/or activity [6,7]. Therefore, many synthetic antioxidants have been studied to prevent food corruption, delay the aging process, and treat degenerative diseases via scavenging ROS [8,9,10,11]. However, there are restrictions on the use of synthetic antioxidants, such as tertiary butylhydroquinone (TBHQ), butylated hydroxyanisole (BHA), butylated hydroxytoluene (BHT) and bisphenol (BP), because they are potentially cytotoxic when used in high concentrations [12].

Phenolic compounds have been extensively studied as antioxidants because they provide a relatively stable free-radical structure after losing a hydrogen atom. Previous reports have shown that the antioxidant activity of phenolic compounds increases upon increasing the number of hydroxyl and methoxy groups on the phenyl ring [13]. Caffeic acid (CA, 3,4-dihydroxycinnamic acid) is a member of the hydroxycinnamic acid (HCA) family and is often found in nature. CA exhibits high antioxidant activity because of the delocalization of non-covalent electrons in its extended side chain [14]. In addition, the *ortho*-dihydroxyl group in CA forms a stable hydrogen bond after dissociation of the O-H bond [15,16,17]. To date, various CA derivatives, such as CA-triazole, CA-tyramine and CA-amino phenol, have been studied. However, such CA derivatives have insufficient redox-active catechol moiety or participate in side reactions, which decrease the antioxidative activity [18,19,20].

**Figure 1:**
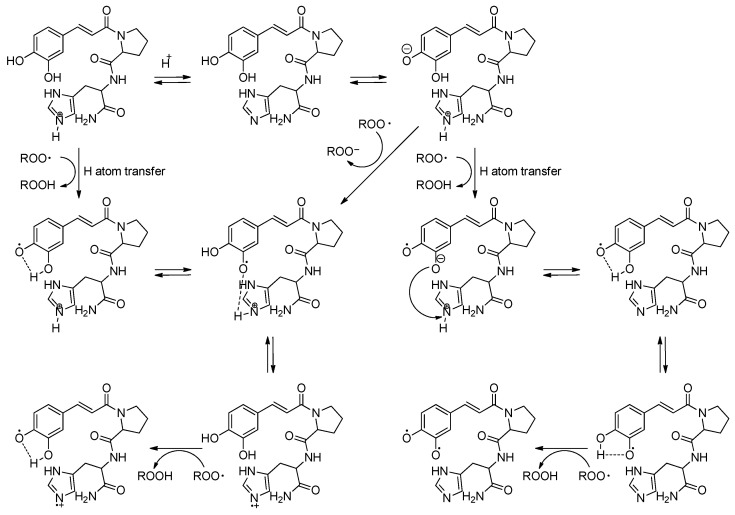
Antioxidant mechanism of CA-PH-NH_2_ with peroxyl radicals based on an H-atom transfer and single-electron transfer mechanism. Reproduced from [26], with permission of Springer Nature Limited.

We have previously reported that the conjugation of CA to a peptide can enhance its stability. In particular, CA-Pro-His-NH_2_ exhibits the highest antioxidant activity reported to date because the s-*cis* proline conformer contributes to the increased antioxidant activity of CA (Figure 1) [21,22,23,24,25,26]. In this study, we expected that some of the CA-tripeptide derivatives would exhibit better antioxidant activity and stability than CA-Pro-His-NH_2_. Therefore, several CA-Pro-His-Xaa-NH_2_ derivatives were synthesized as an extended library from CA-Pro-His-NH_2_, and their antioxidant activity and long-term storage stability were evaluated.

## 2. Results and Discussion

### 2.1. Synthesis of CA-PHX-NH_2_

**Scheme 1:**
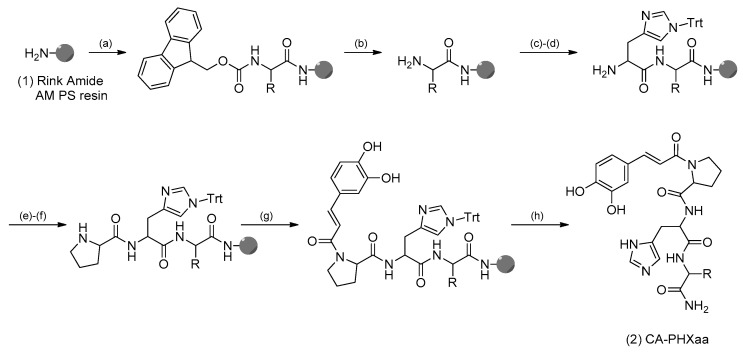
Solid-phase synthesis of the CA-PHX derivatives. Reagents and conditions: (a) Fmoc-L-amino acid (3 equiv.), HBTU (3 equiv.), HOBt (3 equiv.), DIPEA (6 equiv.), DMF, 2 h; (b) 20% piperidine/DMF (*v*/*v*), 5 and 10 min; (c) Fmoc-L-His-OH (3 equiv.), HBTU (3 equiv.), HOBt (3 equiv.), DIPEA (6 equiv.), DMF, 2 h; (d) repeat (b); (e) Fmoc-L-Pro-OH (3 equiv.), HBTU (3 equiv.), HOBt (3 equiv.), DIPEA (6 equiv.), DMF, 2 h; (f) repeat (b); (g) CA (3 equiv.), DIC (3 equiv.), HOBt (3 equiv.), DIPEA (6 equiv.), DMF, 2 h; (h) treatment of dry resin with cleavage cocktail (88% TFA/5% phenol/5% water/2% TIPS) for 1 h and precipitation using diethyl ether. R: side chain of amino acid (serine, arginine, aspartic acid, glycine, asparagine, phenylalanine, lysine, glutamic acid, alanine and glutamine).

Several CA-PHX-NH_2_ derivatives were synthesized on Rink amide aminomethyl polystyrene (AM PS) resin using Fmoc-chemistry-based solid-phase peptide synthesis (SPPS) (Scheme 1). Prior to coupling CA to the peptides on the resin, the purity of the PHX-NH_2_ synthesized on the resin was determined using high-performance liquid chromatography (HPLC) after a small-scale cleavage reaction. The HPLC analysis showed that the purity of PHX-NH_2_ synthesized using Rink amide AM PS resin was >93%. The effect of the resin on the purity of PHX-NH_2_ was evaluated by synthesizing PHX-NH_2_ using Rink amide 4-methylbenzhydrylamine (MBHA) resin, which is known to result in higher purity during SPPS [27]. Unexpectedly, the Rink amide AM PS resin gave an approximately two-fold higher yield than Rink amide MBHA resin, as well as a 3–7% higher crude purity for all the compounds studied (Table 1). The Rink amide MBHA resin released the fragment of Rink amide linker as well as the tripeptide products during the final cleavage step, whereas Rink amide AM PS did not release any compound except the tripeptide products. This affected the purity and yield of the tripeptide products observed during HPLC when the peptides were synthesized on Rink amide MBHA resin.

Thus, several CA-PHX-NH_2_ derivatives were synthesized on Rink amide AM PS resin and obtained as white powders upon precipitation of the cleaved product using cold diethyl ether with crude purities in the range of 72–79%, as confirmed by ESI-MS (Table 2). Finally, the CA-PH-NH_2_ and CA-PHX-NH_2_ derivatives were obtained with >96% purity using preparative HPLC (Supplementary Figures S1–S11). The purified products were used for further investigation of their antioxidant activity.

### 2.2. Antioxidative Activities of the CA-PHX-NH_2_ Derivatives

#### 2.2.1. 2,2-Diphenyl-1-picrylhydrazyl (DPPH) Test

**Figure 2:**
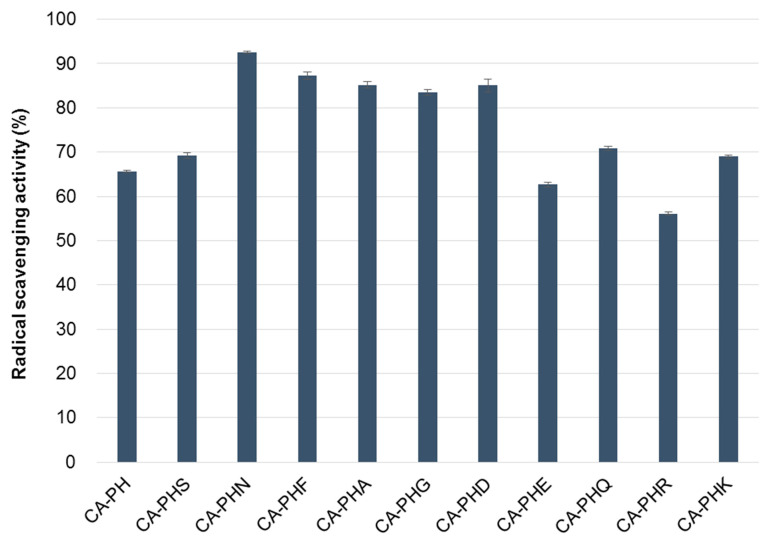
DPPH radical scavenging activity of the CA-PHX-NH_2_ derivatives. Each experiment was performed in triplicate. Results are presented as mean ± standard error.

The free-radical scavenging activity (RSA) of the purified CA-PHX-NH_2_ derivatives was evaluated using a DPPH radical scavenging test. When the CA-PHX-NH_2_ derivatives were in their radical form after donating a hydrogen atom to DPPH, the color of the DPPH solution in methanol (MeOH) changed from purple to yellow (Supplementary Figure S12), and the absorbance observed at 516 nm decreased. Figure 2 shows the observed RSA (%) values, which were calculated using the following equation:

RSA (%) = (1 − Abs. of sample/Abs. of control) × 100.

Some of the CA-PHX-NH_2_ derivatives exhibited higher RSA (%) values than CA-PH-NH_2_, in the following order: CA-PHN-NH_2_ (92.5) > CA-PHF-NH_2_ (87.2) ≈ CA-PHA-NH_2_ (85.2) ≈ CA-PHD-NH_2_ (85.1) ≈ CA-PHG-NH_2_ (83.5) > CA-PHQ-NH_2_ (70.8) ≈ CA-PHS-NH_2_ (69.2) ≈ CA-PHK-NH_2_ (69.0) > CA-PH-NH_2_ (65.5) > CA-PHE-NH_2_ (62.7) > CA-PHR-NH_2_ (56.1). The various side chains of the amino acids influenced the coordination with catechol moiety in CA-PHX-NH_2_, which resulted in different antioxidant activities. CA-PHN-NH_2_ exhibited the highest RSA (%) value when compared to CA-PHX-NH_2_ and CA-PH-NH_2_. The addition of asparagine further stabilized the oxidation state of CA-PH-NH_2_. We presume that the antioxidant activity was improved by the amide side chain of asparagine coordinating precisely with the redox-active catechol moiety in CA-PHN-NH_2_. Further research would clarify the structure of the oxidation state of CA-PHN-NH_2_.

#### 2.2.2. Lipid Peroxidation (LPO) Test

The antioxidant activity was also measured using a lipid peroxidation (LPO) inhibition assay using Tween 20-emulsified linoleic acid (Supplementary Figure S13). The percentage of lipid peroxidation inhibition (%Pi) was calculated using the following equation:

%Pi = (1 − Abs. of sample/Abs. of control) × 100.

**Figure 3:**
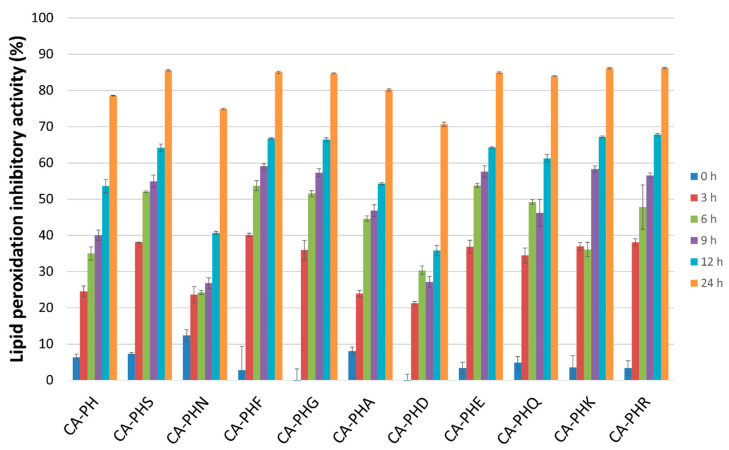
Antioxidant activity of the CA-PHX-NH_2_ derivatives under lipid peroxidation conditions. Each experiment was performed in triplicate. Results are presented as mean ± standard error.

The %Pi values observed for the CA-PHX-NH_2_ derivatives and CA-PH-NH_2_ decreased in the following order after 24 h: CA-PHR-NH_2_ (86.2) > CA-PHK-NH_2_ (86.1) > CA-PHS-NH_2_ (85.5) > CA-PHF-NH_2_ (85.1) > CA-PHE-NH_2_ (85.0) > CA-PHG-NH_2_ (84.7) > CA-PHQ-NH_2_ (84.0) > CA-PHA-NH_2_ (80.2) > CA-PH-NH_2_ (78.6) > CA-PHN-NH_2_ (74.9) > CA-PHD-NH_2_ (70.7). Figure 3 shows that most of the CA-PHX-NH_2_ derivatives were superior to CA-PH-NH_2_ in the LPO inhibition assay. Hydrophilic DPPH and hydrophobic LPO tests showed different patterns for the antioxidant activity among the caffeoyl peptide derivatives studied. For example, CA-PHN-NH_2_ showed the highest antioxidant activity in the DPPH test but had a lower activity than CA-PH-NH_2_ in the LPO inhibition assay. These differences can be attributed to the different solubilities of the derivatives in the working solutions used.

### 2.3. Cytotoxicity of the CA-PHX-NH_2_ Derivatives

**Figure 4:**
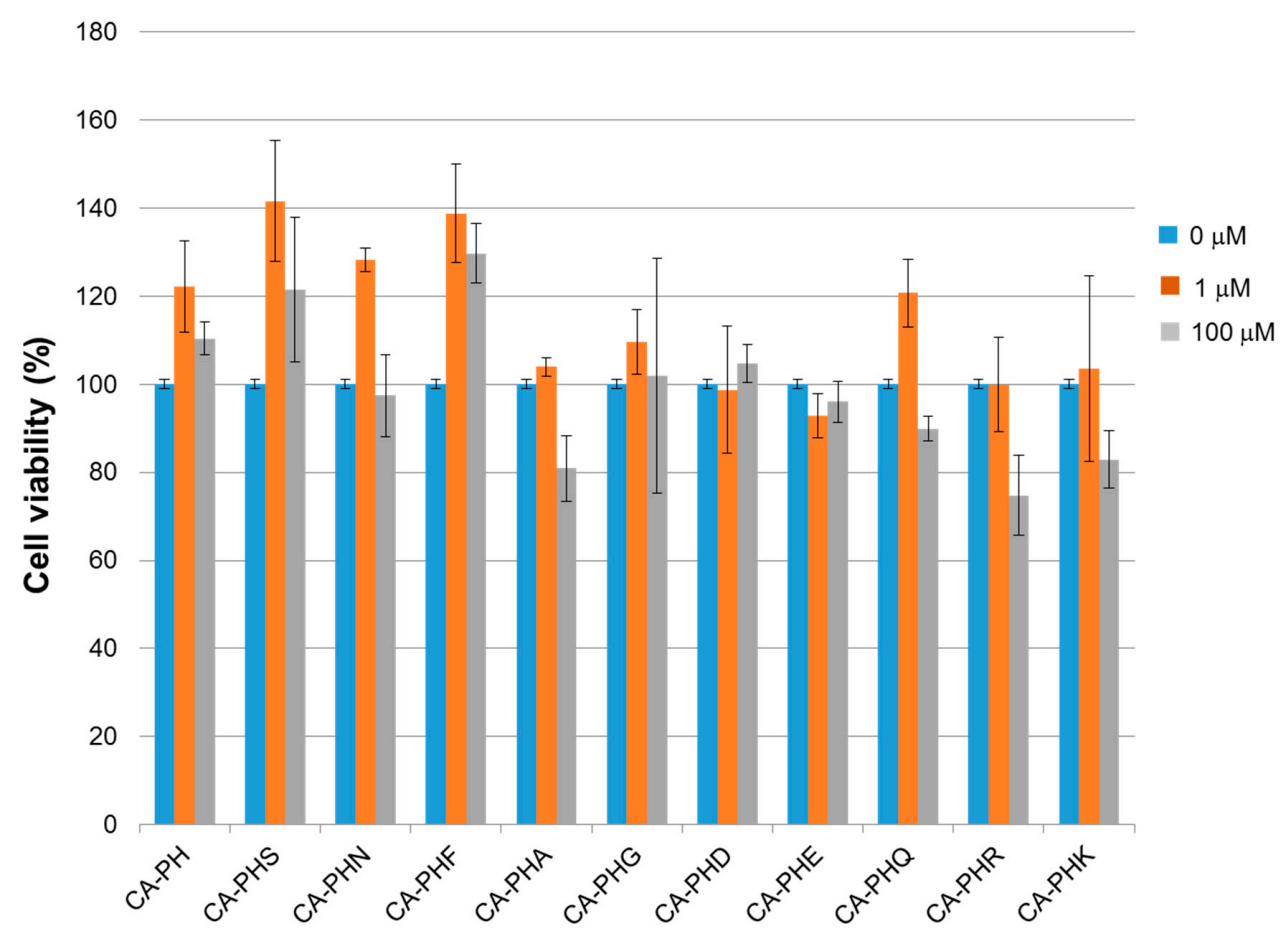
Cytotoxicity of the CA-PHX-NH_2_ derivatives measured using MTT assay. Each experiment was performed in triplicate. Results are presented as mean ± standard error.

The MTT assay data exhibited no significant cytotoxicity for the CA-PHX-NH_2_ derivatives at low concentration (1 µM). Even at concentrations as high as 100 µM, most of the CA-PHX-NH_2_ derivatives were not cytotoxic, while some of the compounds, such as CA-PHA-NH_2_, CA-PHR-NH_2_ and CA-PHK-NH_2_, showed cell viabilities of <80%. In the case of CA-PH-NH_2_, CA-PHS-NH_2_, CA-PHN-NH_2_ and CA-PHQ-NH_2_, cell viabilities exceeded 100%. We presume that those CA derivatives reduced the oxidative stress of the cells and promoted cell proliferation, overcoming cytotoxicity (Figure 4). Thus, CA-PHX-NH_2_ derivatives exhibiting low cytotoxicity have potential use as cosmetic ingredients.

### 2.4. Stability Evaluation Using ^1^H-NMR Spectroscopy

^1^H-NMR spectroscopy was used to evaluate the long-term structural stability of CA-PHN-NH_2_. The changes in the molar ratio of s*-cis* Pro, which demonstrates the stability of CA-PHN-NH_2_, were monitored upon storage at 25 °C under dark conditions over 3 months. The ratio of the s*-cis* form remained >93% for 3 months (Table 3, Supplementary Figure S14). The ^1^H-NMR spectra show that CA-PHN-NH_2_ exhibited limited transition between the *cis* and *trans* conformations over 3 months, which is critical for maintaining its high antioxidation activity [26]. Therefore, CA-PHN-NH_2_ will likely maintain its antioxidant activity in hydrophilic solutions upon long-term storage.

## 3. Materials and Methods

### 3.1. Materials

Triisopropylsilane (TIPS), caffeic acid (CA), 2,2-diphenyl-1-picrylhydrazyl (DPPH), linoleic acid, thiazolyl blue tetrazolium bromide (MTT), ammonium thiocyanate, Tween 20, sodium phosphate dibasic dodecahydrate, sodium phosphate monobasic dihydrate, ethanol (EtOH) and iron(II) chloride tetrahydrate (FeCl_2_·4H_2_O) were purchased from Sigma-Aldrich (St. Louis, MO, USA). Functionalized resins, 9-fluorenylmethyloxycarbonyl (Fmoc)-Rink amide linker, Fmoc amino acids, *N*,*N*′-diisopropylcarbodiimde (DIC), *N*,*N*,*N*′,*N*′-tetramethyl-*O*-(1*H*-benzotriazol-1-yl)uranium hexafluorophosphate (HBTU), 1-hydroxybenzotriazole (HOBt) and filtered reaction tubes (Libra tube^®^) were purchased from BeadTech Inc. (Ansan, Korea). Acetonitrile (ACN), dichloromethane (DCM), *N,N-*diisopropylethylamine (DIPEA), methanol (MeOH), *N*,*N*-dimethylformamide (DMF), dimethylsulfoxide (DMSO), acetic anhydride, hydrochloric acid (HCl), piperidine, trifluoroacetic acid (TFA) and phenol were purchased from Daejung Chemical & Metals Co. Ltd. (Siheung, Korea). NIH 3T3 mouse fibroblasts were purchased from Korea Cell Line Bank (Seoul, Korea) and cultured in 10% (*v*/*v*) fetal bovine serum (FBS) and 1% (*v*/*v*) penicillin streptomycin in RPMI 1640 media (Biowest, Nuaillé, France) at 37 °C in a humidified 5% CO_2_ atmosphere. Trypsin-ethylenediaminetetraacetic acid (EDTA) in Hanks’ balanced salt solution (HBSS) was purchased from Biowest (Nuaillé, France).

### 3.2. Solid-Phase Synthesis of CA-PHX-NH_2_

Prolyl-histidyl-Xaa-NH_2_ was synthesized on two types of resin using Fmoc/*t*-Bu chemistry. The tripeptides were synthesized on a Rink amide resin (300 mg) by repeating the coupling and deprotection steps: (a) in the coupling step, the resins were treated with a solution of Fmoc-amino acid (3.0 equiv.), HBTU (3.0 equiv.), HOBt (3.0 equiv.) and DIPEA (6.0 equiv.) in DMF (10 mL) for 2 h; (b) in the deprotection step, each resin was treated twice with 20% piperidine/DMF (*v*/*v*) solution (2 × 10 mL) for 5 and 10 min. The resins were washed with DMF, DCM and MeOH three times after each coupling and deprotection step. The completion of each amino acid coupling reaction used in the synthesis was confirmed using Kaiser’s ninhydrin test.

The CA (3.0 equiv.) was dissolved in DMF (3 mL) in a glass vial. To this solution, HOBt (3.0 equiv.) and DIC (3.0 equiv.) were added, and the resulting mixture was stirred at room temperature for 30 min under dark conditions. The mixture was added to a reaction tube containing Pro-His-Xaa-NH_2_ and DIPEA (6.0 equiv.) and incubated in a multirotator for 1 h at room temperature. The resin was washed three times with DMF, DCM and MeOH. The resin was treated with a cleavage cocktail comprising trifluoroacetic acid/phenol/triisopropylsilane/water (88:5:5:2, *v*/*v*/*v*/*v*; 3 mL) for 2 h. After removal of the resin, the resulting solution was precipitated upon the addition of diethyl ether. The supernatant was decanted after centrifugation, and the remaining pellet was washed three times with diethyl ether via centrifugation and dried in vacuo. The purities of the peptides and CA-PHX-NH_2_ were analyzed using HPLC under the following conditions: gradient elution with A (0.1% TFA in water) and B (0.1% TFA in acetonitrile) from 10% to 90% B over 41 min; flow rate of 1.0 mL/min; UV detection at 230 and 214 nm; column temperature of 35.0 °C.

### 3.3. Antioxidant Activities of CA-PHX-NH_2_

#### 3.3.1. 2,2-Diphenyl-1-picrylhydrazyl (DPPH) Test

A DPPH/MeOH solution (0.1 mM, 1.48 mL) was placed in a microtube and treated with a solution of the CA-PHX-NH_2_ derivative in MeOH (50 µM, 0.2 mL) for 10 min. The absorbance of the DPPH mixture was measured at 516 nm using a UV/Vis spectrophotometer [22].

#### 3.3.2. LPO Test

A linoleic acid emulsion (25 mM) was prepared by mixing linoleic acid (142 mg), Tween 20 (142 mg) and sodium phosphate buffer (0.1 M, pH 7.0, 50 mL). As antioxidants of the linoleic acid emulsion, solutions of the CA-PHX-NH_2_ derivatives (9, 45 and 90 μM and 0.5 mL) were prepared in a mixture of water (0.5 mL) and sodium phosphate buffer (0.1 M, 2.0 mL) in separate vials for the LPO test. Each mixture was kept at 50 °C under dark conditions for 24 h [28,29]. Each mixture (25 μL) was mixed with 75% EtOH (1.175 mL), FeCl_2_·4H_2_O in 3.5% hydrochloric acid (HCl) (20 mM, 25 μL) and 25 μL of 30% NH_4_SCN for 3 min. The absorbance of the mixtures at 500 nm was measured using a UV/Vis spectrophotometer.

### 3.4. Cytotoxicity Assay for CA-PHX-NH_2_

NIH 3T3 cells were seeded (100,000 cells/mL, 200 μL) in a 96-well plate and incubated for 1 day. Solutions of each CA-PHX-NH_2_ derivative in RPMI 1640 media (1, 10, 50 and 100 μM) were prepared. The cell culture medium in each well was carefully aspirated, and the cells were treated with the CA-PHX-NH_2_ solutions for 1 day in a CO_2_ incubator. After removal of the solution, the cells were incubated in the presence of MTT solution (200 μL) for 3 h. After incubation, the MTT solution was discarded and DMSO was added to dissolve the formazan produced by the living cells. The mixtures in the 96-well plate were shaken in a shaking incubator for 20 min. The absorbance of the dissolved formazan was measured at a wavelength of 590 nm using a microplate reader. The percentage of cell viability was calculated by comparison with the number of standard cells which were not treated with the solutions of CA derivatives.

### 3.5. Long-Term Stability Test Using ^1^H-NMR Spectroscopy

The sample of CA-PHN-NH_2_ for ^1^H-NMR spectroscopy was prepared by dissolving 10 mg of compound (±0.01 mg) in 1 mL of methanol-d_6_ (MeOD). The resulting solution was transferred to a 5 mm standard NMR tube. The ^1^H-NMR spectra were obtained on a Bruker AVANCE III-HD500 spectrometer operated at 500 MHz. Tetramethylsilane (TMS) was used as an internal standard for the analysis of the chemical shifts.

## 4. Conclusions

A library of caffeoyl tripeptides (CA-PHX-NH_2_) was synthesized, and their antioxidant activities were evaluated to develop new CA-peptide derivatives with enhanced antioxidant capacity compared to CA-PH-NH_2_. The DPPH assay showed that CA-PHN-NH_2_ exhibited the highest free RSA (92.5%), compared to CA-PHX-NH_2_ and CA-PH-NH_2_. None of the CA derivatives exhibited significant cytotoxicity at concentrations up to 100 µM. ^1^H-NMR spectroscopy showed that CA-PHN-NH_2_ maintained its structural integrity for up to 3 months. Therefore, CA-PHN-NH_2_ is expected to be used as a raw material for pharmaceutical or cosmetic ingredients because of its superior antioxidant ability and long-term stability.

## Figures and Tables

**Table 1:** Yields and purities of the representative tripeptides (PHX-NH_2_) synthesized using Rink amide MBHA resin and Rink amide AM PS resin.

Tripeptide	Rink Amide MBHA Resin		Rink Amide AM PS Resin	
	Yield (%)	Purity (%)	Yield (%)	Purity (%)
PHS-NH_2_	49.4	89.7	89.6	96.2
PHA-NH_2_	57.5	94.0	82.2	97.4
PHN-NH_2_	49.5	90.0	>99	95.4
PHD-NH_2_	47.8	89.5	97.3	93.7

**Table 2:** Purity and ESI-MS results of CA-PHX-NH_2_.

CA-PHX-NH_2_	Crude Purity (%)	Refinement Purity (%)	ESI-MS	
			Calculated	Found
			[M + H]^+^	[M + H]^+^
CA-PH-NH_2_	74.6	98.2	414.43	414.02
CA-PHS-NH_2_	77.6	98.5	501.49	501.07
CA-PHR-NH_2_	76.3	97.4	570.60	570.00
CA-PHD-NH_2_	72.6	99.5	529.5	529.04
CA-PHG-NH_2_	73.4	97.1	471.46	471.03
CA-PHN-NH_2_	76.9	98.7	528.51	528.09
CA-PHF-NH_2_	79.9	99.2	561.59	561.02
CA-PHK-NH_2_	79.7	98.5	542.58	542.14
CA-PHE-NH_2_	75.9	97.7	543.53	543.04
CA-PHA-NH_2_	73.4	98.1	485.49	485.07
CA-PHQ-NH_2_	75.1	96.1	542.54	541.96

**Table 3:** Ratio of the s*-cis* form of CA-PHN-NH_2_.

CA-PHN-NH_2_	Relative Integral Intensity (%)		
	(Ratio of *s-cis* Form)		
	CH= Proton	His C^ε1^-H Proton	Average
0 h	94.38	94.61	94.50
1 week	93.84	94.01	93.93
1 month	93.40	94.22	93.81
2 months	95.04	93.82	94.43
3 months	93.69	95.53	94.61

## Supplementary Materials

Supplementary materials can be found at https://www.mdpi.com/article/10.3390/ijms22126301/s1. Figure S1. HPLC chromatograms of (a) crude CA-PH-NH_2_, (b) purified CA-PH-NH_2_, and (c) ESI-MS data of CA-PH-NH_2_, Figure S2. HPLC chromatograms of (a) crude CA-PHS-NH_2_, (b) purified CA-PHS-NH_2_, and (c) ESI-MS data of CA-PHS-NH_2_, Figure S3. HPLC chromatograms of (a) crude CA-PHR-NH_2_, (b) purified CA-PHR-NH_2_, and (c) ESI-MS data of CA-PHR-NH_2_, Figure S4. HPLC chromatograms of (a) crude CA-PHD-NH_2_, (b) purified CA-PHD-NH_2_, and (c) ESI-MS data of CA-PHD-NH_2_, Figure S5. HPLC chromatograms of (a) crude CA-PHG-NH_2_, (b) purified CA-PHG-NH_2_, and (c) ESI-MS data of CA-PHG-NH_2_, Figure S6. HPLC chromatograms of (a) crude CA-PHN-NH_2_, (b) purified CA-PHN-NH_2_, and (c) ESI-MS data of CA-PHN-NH_2_, Figure S7. HPLC chromatograms of (a) crude CA-PHF-NH_2_, (b) purified CA-PHF-NH_2_, and (c) ESI-MS of CA-PHF-NH_2_, Figure S8. HPLC chromatograms of (a) crude CA-PHK-NH_2_, (b) purified CA-PHK-NH_2_, and (c) ESI-MS of CA-PHK-NH_2_, Figure S9. HPLC chromatograms of (a) crude CA-PHE-NH_2_, (b) purified CA-PHE-NH_2_, and (c) ESI-MS data of CA-PHE-NH_2_, Figure S10. HPLC chromatograms of (a) crude CA-PHA-NH_2_, (b) purified CA-PHA-NH_2_, and (c) ESI-MS data of CA-PHA-NH_2_, Figure S11. HPLC chromatograms of (a) crude CA-PHQ-NH_2_, (b) purified CA-PHQ-NH_2_, and (c) ESI-MS data of CA-PHQ-NH_2_, Figure S12. Mechanism of the DPPH radical scavenging test, Figure S13. Mechanism of lipid peroxidation inhibition test, Figure S14. ^1^H-NMR spectra of CA-PHN-NH_2_ tripeptides for 3 months at room temperature in the dark.

## Abbreviations

CA-PHX-NH_2_	Caffeoyl-prolyl-histidyl-X amino acid amide
AM PS	Aminomethyl polystyrene
MBHA	4-Methylbenzhydrylamine
DPPH	2,2-Diphenyl-1-picrylhydrazyl
LPO	Lipid peroxidation
