# Supplementary material for: Synthesis of Caffeoyl-Prolyl-Histidyl-Xaa Derivatives and Evaluation of Their Activities and Stability upon Long-Term Storage
Source: Int J Mol Sci. 2021 Jun 11;22(12):6301. doi: 10.3390/ijms22126301 (PMC8231216; doi:10.3390/ijms22126301)
Supplement: Supplementary file 1 [file ijms-22-06301-s001.zip › ijms-1249259-supplementary.pdf]

International Journal of
*Molecular Sciences*

MDPI

# Synthesis of Caffeoyl-Prolyl-Histidyl-Xaa Derivatives and Evaluation of Their Activities and Stability upon Long-Term Storage

**Hyeri Jeong[1,†], Young-Eun Jeon[1,†], Jin-Kyoung Yang[2], Jaehi Kim[2], Woo-Jae Chung[3], Yoon-Sik Lee[2,4*] and Dong-Sik Shin[1,5*]**

[1] Department of Chemical and Biological Engineering, Sookmyung Women's University, Seoul 04310, Korea
[2] School of Chemical and Biological Engineering, Seoul National University, Seoul 08826, Korea
[3] Department of Integrative Biotechnology, Sungkyunkwan University, Suwon 16419, Republic of Korea
[4] BeadTech Inc., 49 Wonsi-ro, Danwon-gu, Ansan-si, Gyeonggi-do 15610, Korea
[5] Industry Collaboration Center, Sookmyung Women's University, Seoul 04310, Korea
†These authors contributed equally to the work
* Correspondence: Correspondence: yslee@beadtech.co.kr (Y.-S.L.); dshin@sookmyung.ac.kr (D.-S.S.); Tel.: +82-31-8084-8359 (Y.-S.L.), +82-2-2077-7236 (D.-S.S.)

a)

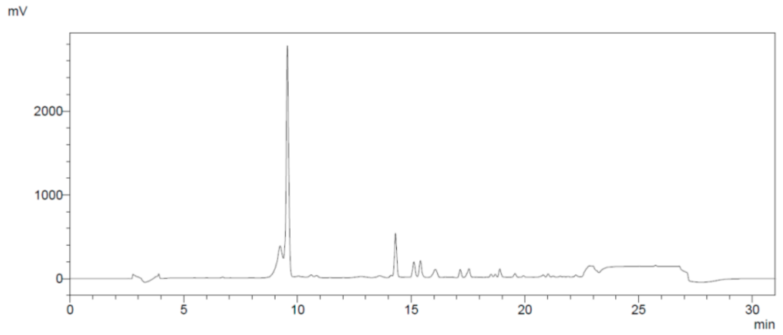


b)

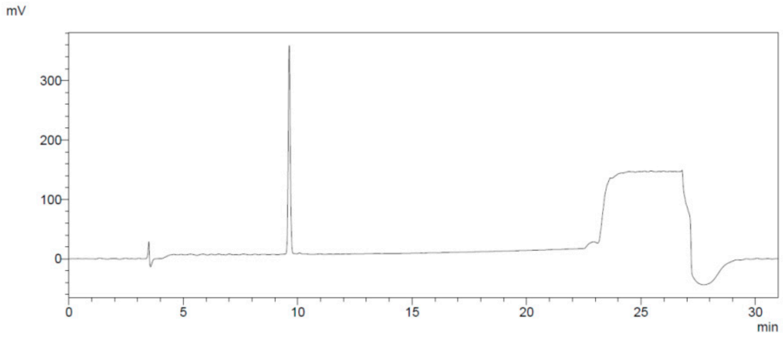


c)

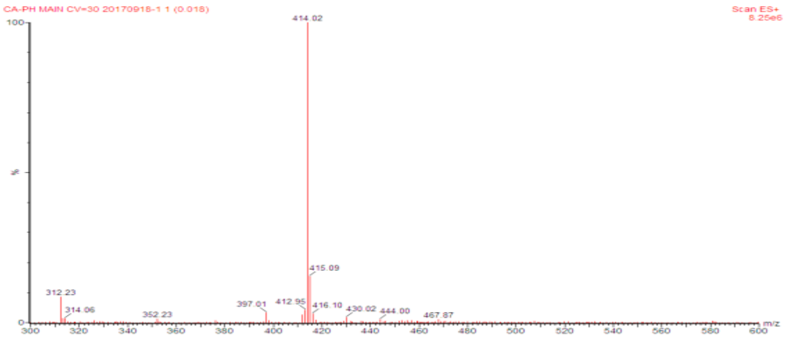


**Figure S1.** HPLC chromatogram of a) crude CA-PH-$NH_2$, b) purified CA-PH-$NH_2$, and c) ESI-MS data of CA-PH-$NH_2$.

a)

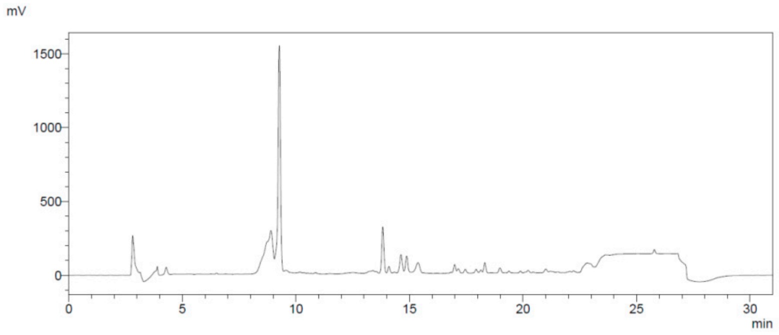


b)

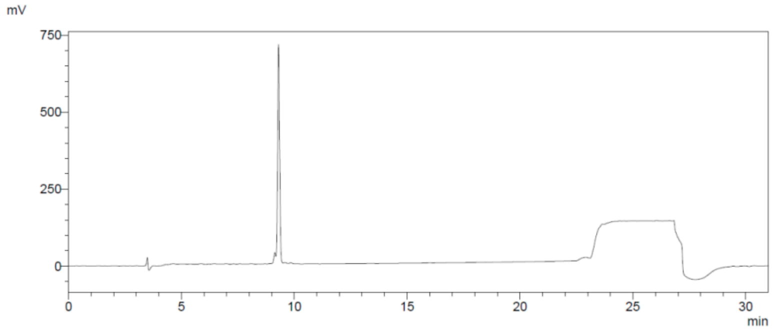


c)

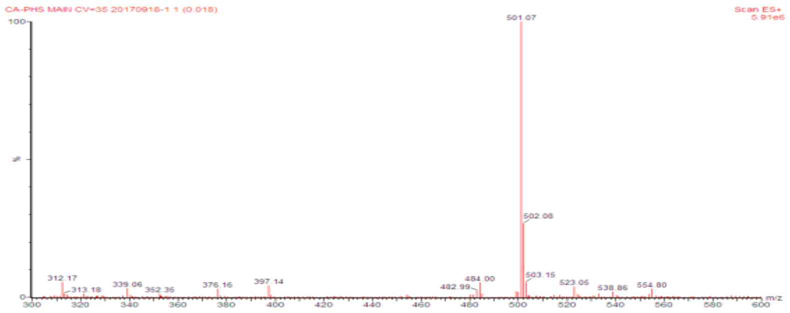


**Figure S2.** HPLC chromatogram of a) crude CA-PHS-$NH_2$, b) purified CA-PHS-$NH_2$, and c) ESI-MS data of CA-PHS-$NH_2$.

a)

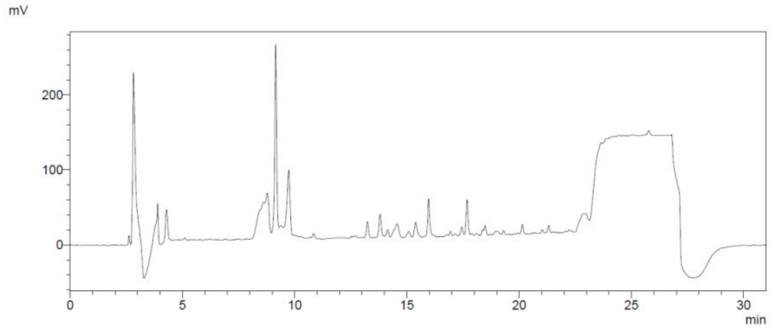


b)

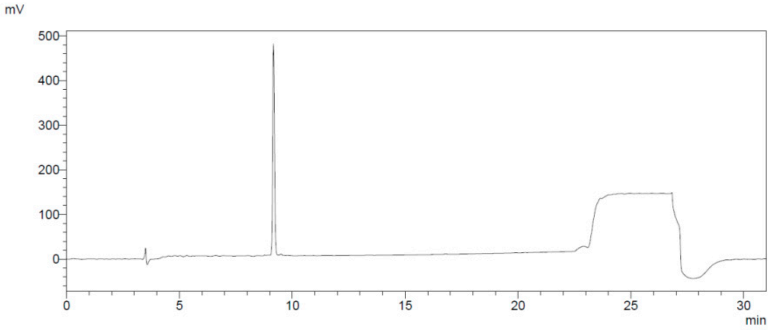


c)

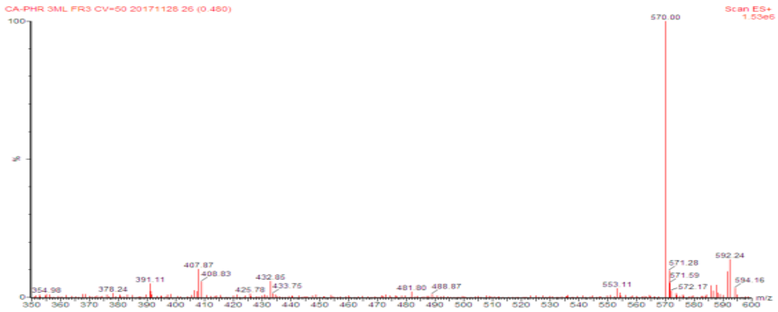


**Figure S3.** HPLC chromatogram of a) crude CA-PHR-$NH_2$, b) purified CA-PHR-$NH_2$, and c) ESI-MS data of CA-PHR-$NH_2$.

a)

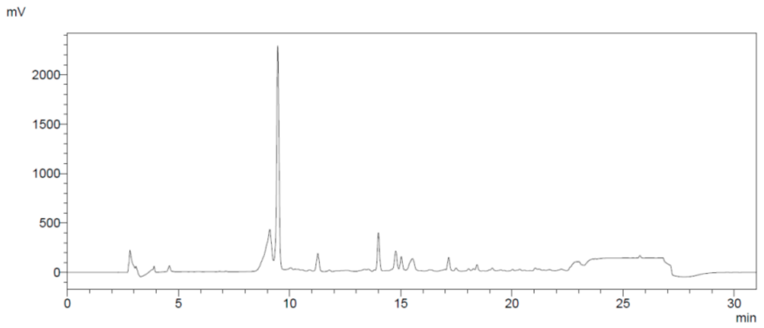


b)

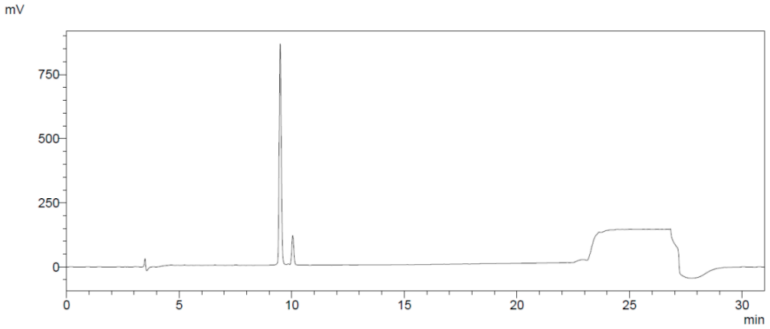


c)

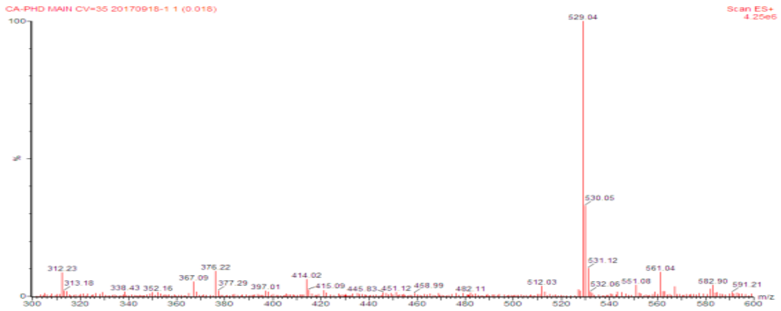


**Figure S4.** HPLC chromatogram of a) crude CA-PHD-$NH_2$, b) purified CA-PHD-$NH_2$, and c) ESI-MS data of CA-PHD-$NH_2$.

a)

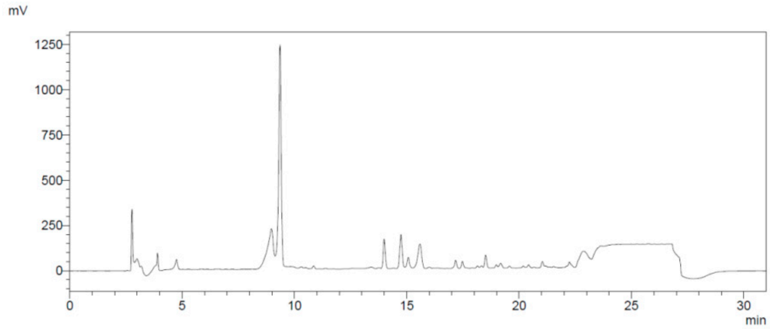


b)

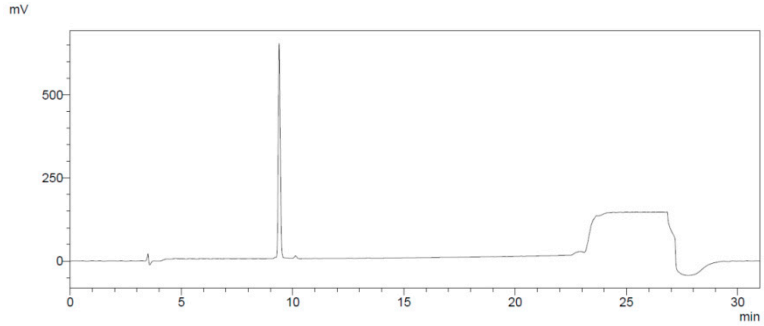


c)

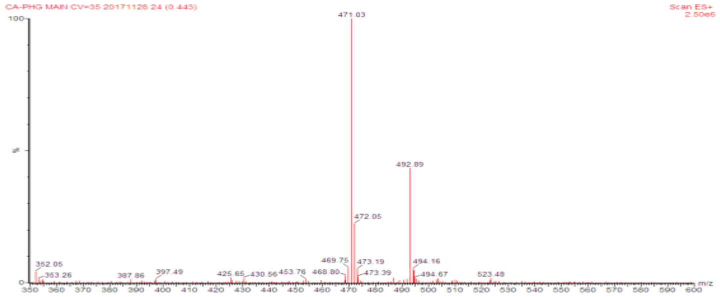


**Figure S5.** HPLC chromatogram of a) crude CA-PHG-$NH_2$, b) purified CA-PHG-$NH_2$, and c) ESI-MS data of CA-PHG-$NH_2$.

a)

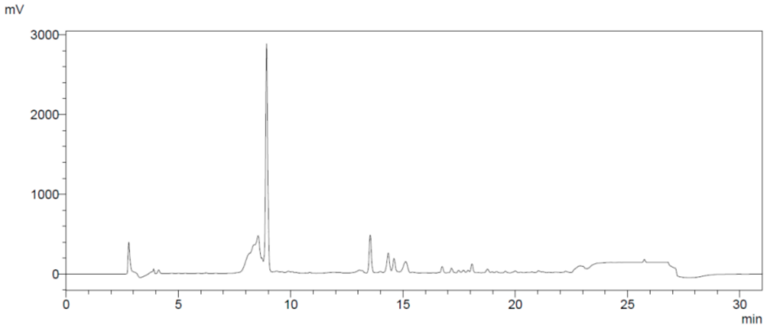


b)

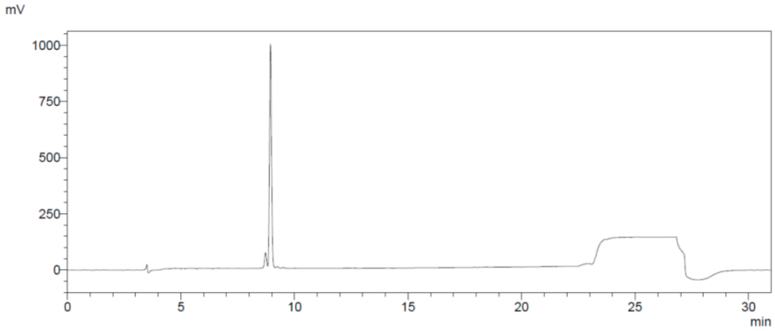


c)

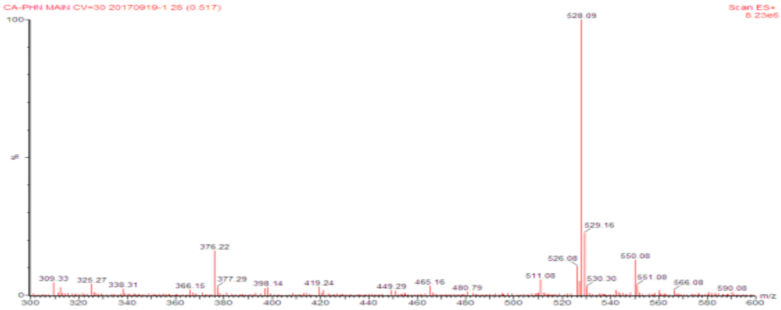


**Figure S6.** HPLC chromatogram of a) crude CA-PHN-$NH_2$, b) purified CA-PHN-$NH_2$, and c) ESI-MS data of CA-PHN-$NH_2$.

a)

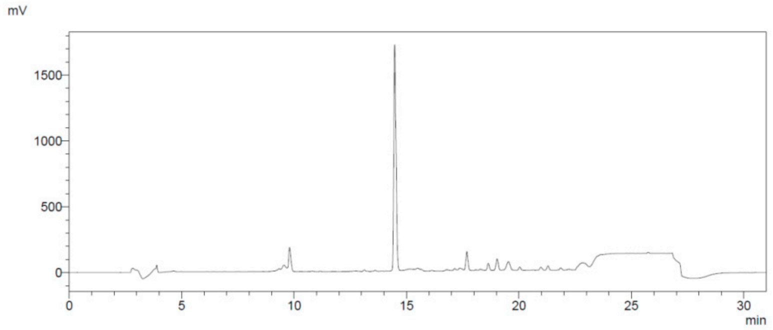


b)

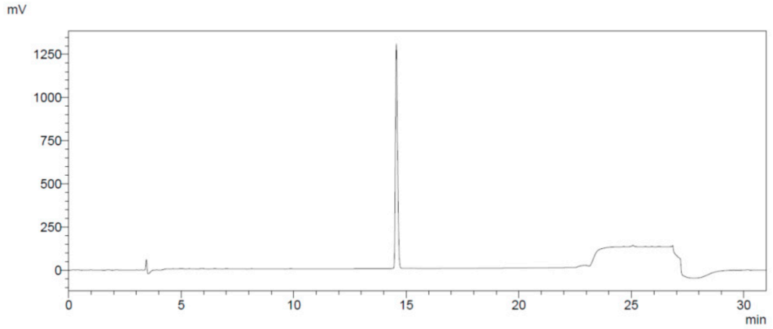


c)

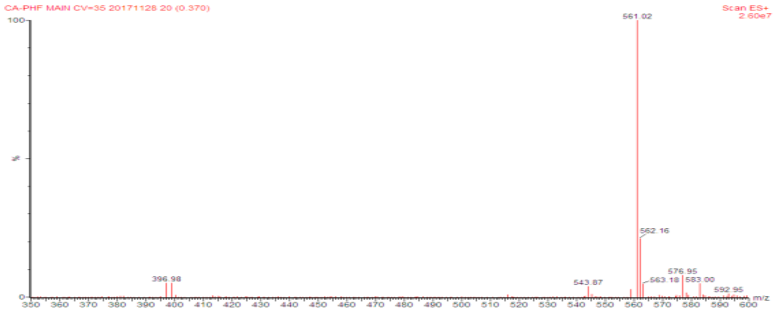


**Figure S7.** HPLC chromatogram of a) crude CA-PHF-$NH_2$, b) purified CA-PHF-$NH_2$, and c) ESI-MS of CA-PHF-$NH_2$.

a)

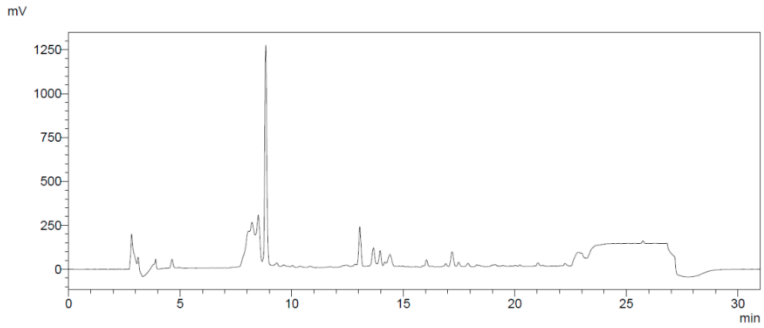


b)

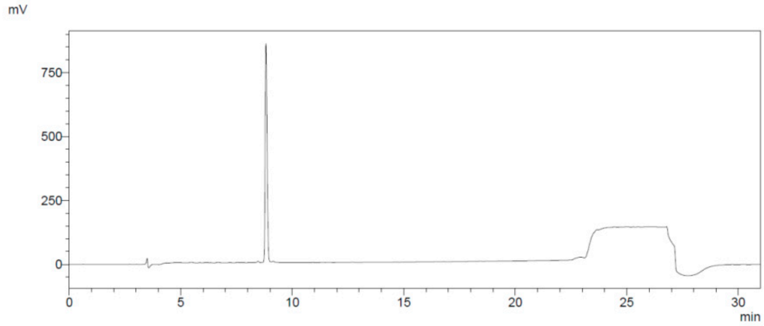


c)

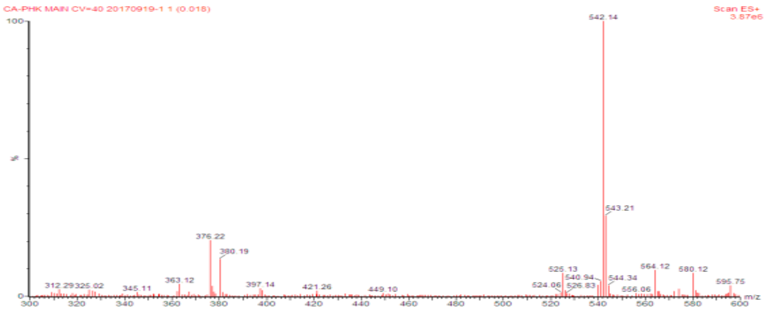


**Figure S8.** HPLC chromatogram of a) crude CA-PHK-$NH_2$, b) purified CA-PHK-$NH_2$, and c) ESI-MS of CA-PHK-$NH_2$.

a)

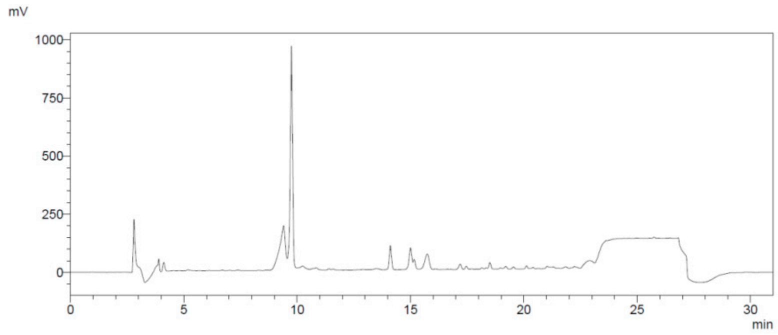


b)

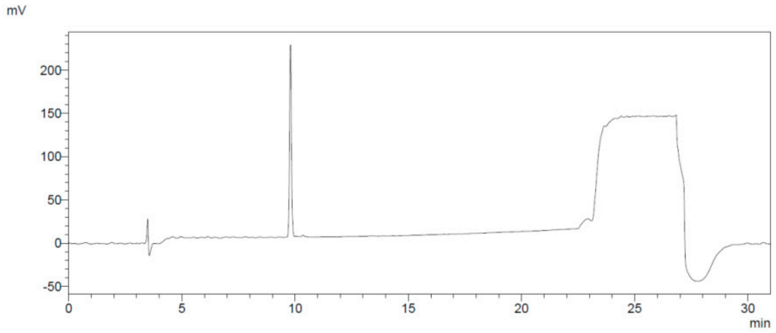


c)

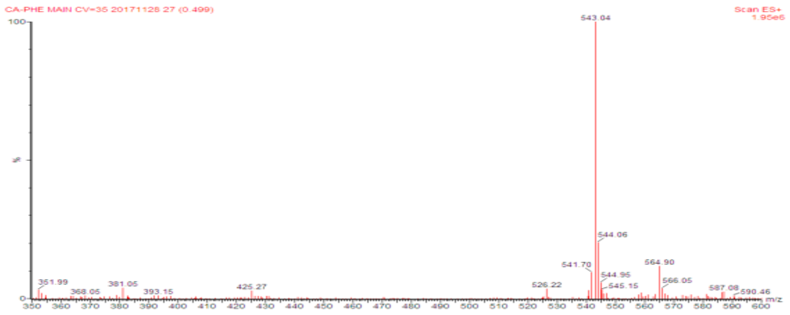


**Figure S9.** HPLC chromatogram of a) crude CA-PHE-$NH_2$, b) purified CA-PHE-$NH_2$, and c) ESI-MS data of CA-PHE-$NH_2$.

a)

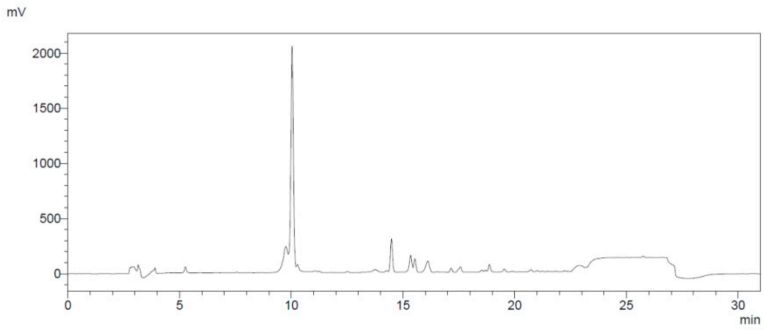


b)

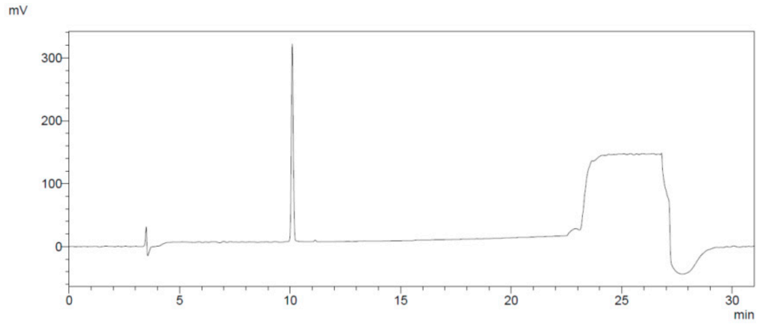


c)

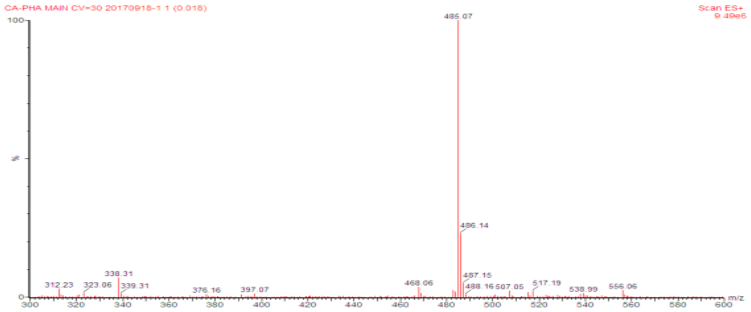


**Figure S10.** HPLC chromatogram of a) crude CA-PHA-$NH_2$, b) purified CA-PHA-$NH_2$, and c) ESI-MS data of CA-PHA-$NH_2$.

a)

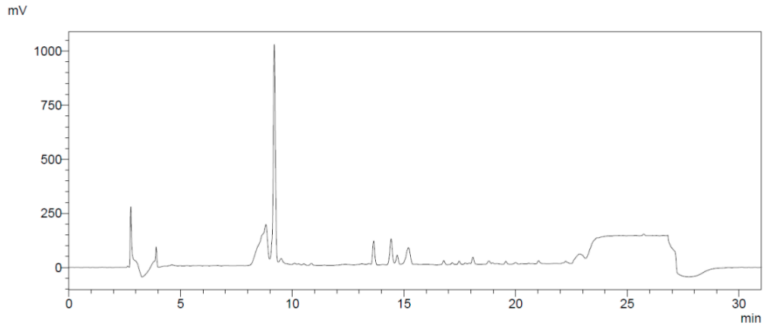


b)

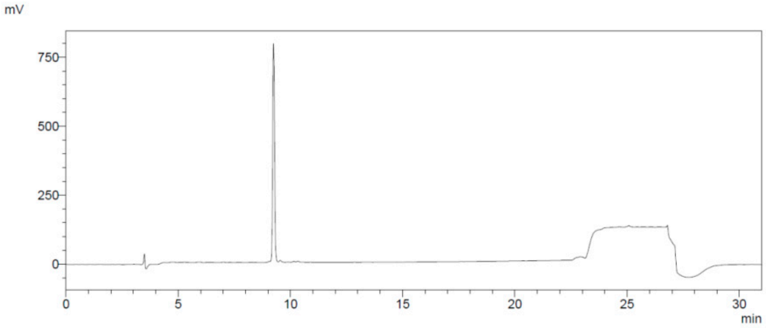


c)

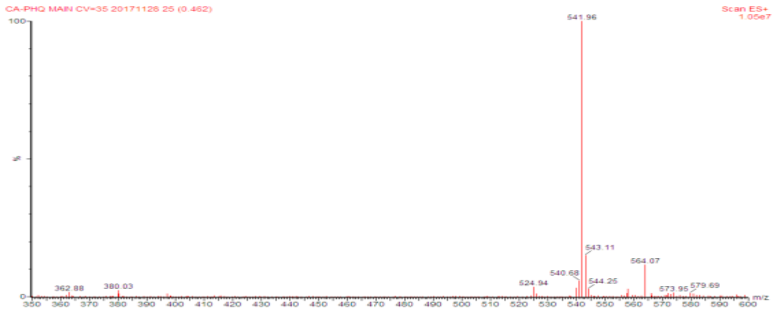


**Figure S11.** HPLC chromatogram of a) crude CA-PHQ-$NH_2$, b) purified CA-PHQ-$NH_2$, and c) ESI-MS data of CA-PHQ-$NH_2$.

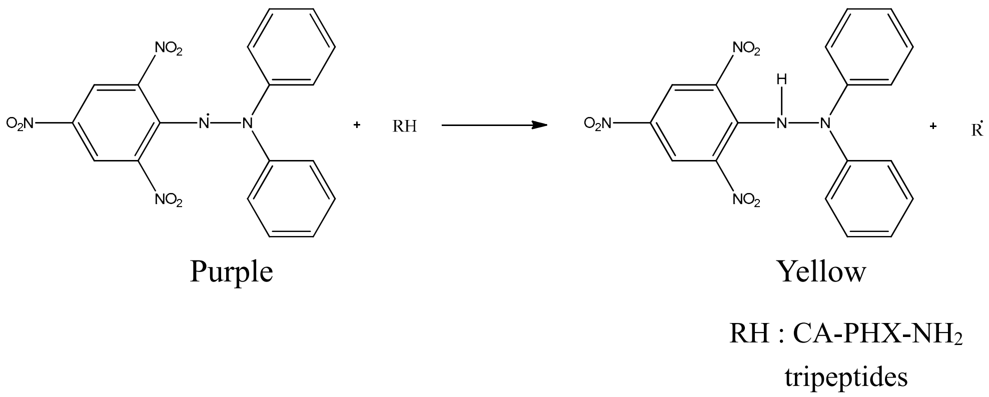


**Figure S12.** Mechanism of DPPH radical scavenging test.

.

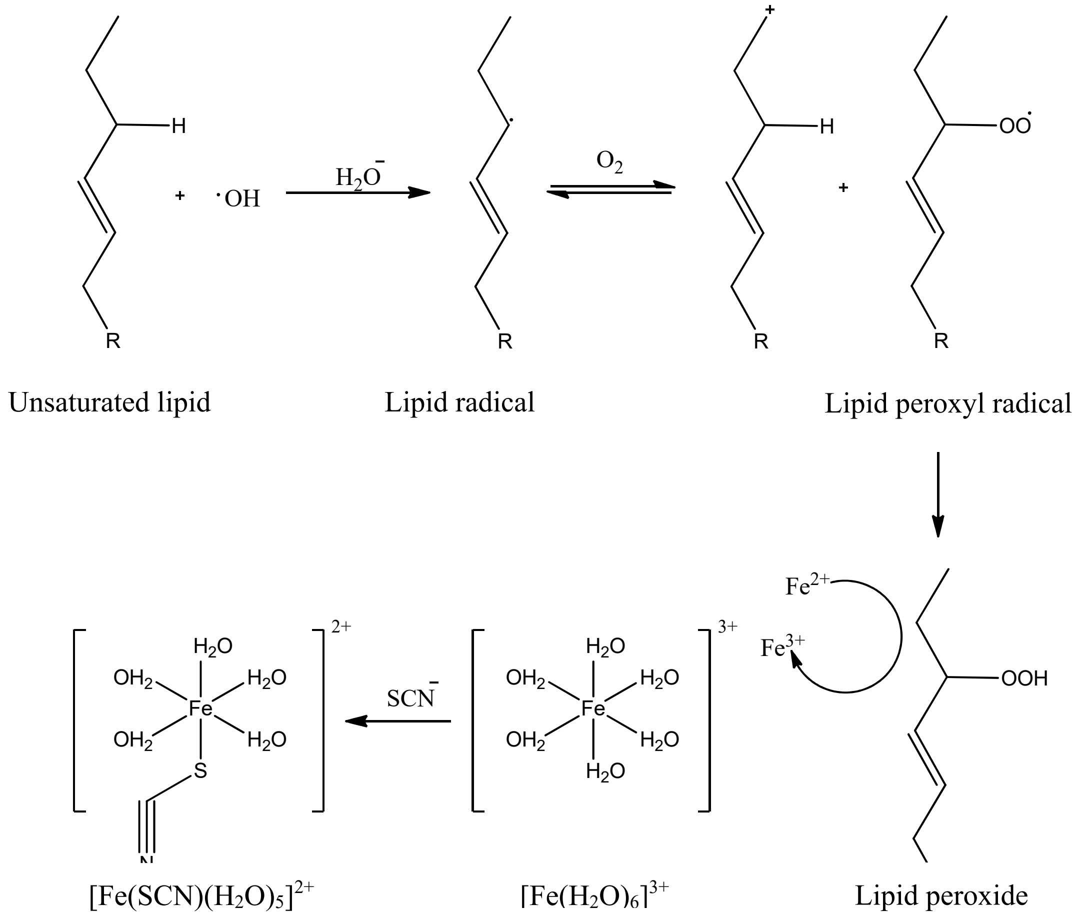


**Figure S13.** Mechanism of lipid peroxidation inhibition test.

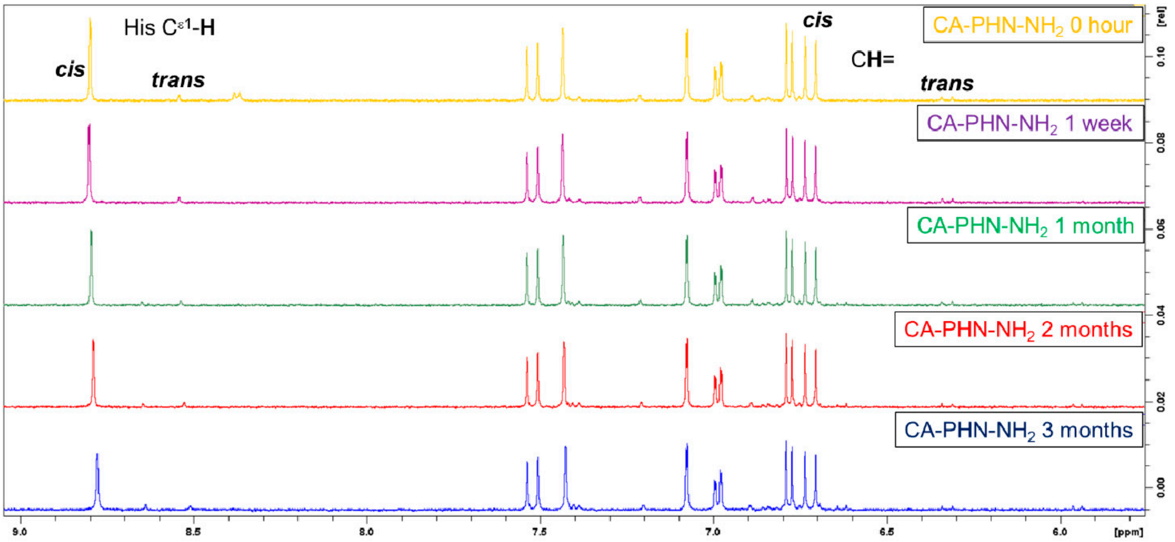


**Figure S14.** $^1$H-NMR spectrum of CA-PHN-$NH_2$ tripeptides for 3 months at room temperature in the dark room.
